# A Metagenomic Approach to Characterization of the Vaginal Microbiome Signature in Pregnancy

**DOI:** 10.1371/journal.pone.0036466

**Published:** 2012-06-13

**Authors:** Kjersti Aagaard, Kevin Riehle, Jun Ma, Nicola Segata, Toni-Ann Mistretta, Cristian Coarfa, Sabeen Raza, Sean Rosenbaum, Ignatia Van den Veyver, Aleksandar Milosavljevic, Dirk Gevers, Curtis Huttenhower, Joseph Petrosino, James Versalovic

**Affiliations:** 1 Department of Obstetrics & Gynecology, Division of Maternal-Fetal Medicine, Baylor College of Medicine and Texas Children’s Hospital, Houston, Texas, United States of America; 2 Department of Pathology & Immunology, Baylor College of Medicine and Texas Children’s Hospital, Houston, Texas, United States of America; 3 Bioinformatics Research Laboratory, Baylor College of Medicine and Texas Children’s Hospital, Houston, Texas, United States of America; 4 Harvard School of Public Health, Harvard University, Cambridge, Massachusetts, United States of America; 5 Broad Institute, Massachusetts Institute of Technology, Cambridge, Massachusetts, United States of America; 6 Department of Molecular Virology and Microbiology, Baylor College of Medicine and Texas Children’s Hospital, Houston, Texas, United States of America; Columbia University, United States of America

## Abstract

While current major national research efforts (*i.e.,* the NIH Human Microbiome Project) will enable comprehensive metagenomic characterization of the adult human microbiota, how and when these diverse microbial communities take up residence in the host and during reproductive life are unexplored at a population level. Because microbial abundance and diversity might differ in pregnancy, we sought to generate comparative metagenomic signatures across gestational age strata. DNA was isolated from the vagina (introitus, posterior fornix, midvagina) and the V5V3 region of bacterial 16S rRNA genes were sequenced (454FLX Titanium platform). Sixty-eight samples from 24 healthy gravidae (18 to 40 confirmed weeks) were compared with 301 non-pregnant controls (60 subjects). Generated sequence data were quality filtered, taxonomically binned, normalized, and organized by phylogeny and into operational taxonomic units (OTU); principal coordinates analysis (PCoA) of the resultant beta diversity measures were used for visualization and analysis in association with sample clinical metadata. Altogether, 1.4 gigabytes of data containing >2.5 million reads (averaging 6,837 sequences/sample of 493 nt in length) were generated for computational analyses. Although gravidae were not excluded by virtue of a posterior fornix pH >4.5 at the time of screening, unique vaginal microbiome signature encompassing several specific OTUs and higher-level clades was nevertheless observed and confirmed using a combination of phylogenetic, non-phylogenetic, supervised, and unsupervised approaches. Both overall diversity and richness were reduced in pregnancy, with dominance of Lactobacillus species (L. *iners crispatus*, *jensenii* and *johnsonii*, and the orders Lactobacillales (and *Lactobacillaceae* family), Clostridiales, Bacteroidales, and Actinomycetales. This intergroup comparison using rigorous standardized sampling protocols and analytical methodologies provides robust initial evidence that the vaginal microbial 16S rRNA gene catalogue uniquely differs in pregnancy, with variance of taxa across vaginal subsite and gestational age.

## Introduction

To date, the dominant paradigm in Western medicine considers microbes as “foreign” and has led to the prevailing view that elimination of predominant pathogens will result in amelioration of disease. Such a view is seemingly in contrast to longstanding observations that humans serve as host to co-evolving microbes residing in highly plethoric communities. Indeed, microbiota are present from the time of birth, with up to 10-fold the number of microorganisms to adult human cells and a collective genome (the “metagenome”) which exceeds our human genome in terms of gene content by more than 100-fold [1]. Moreover, we appreciate that the human microbiota are a metabolically and antigenically vibrant and diverse community which may function as *mutualists* (symbiotically beneficial), *commensals* (of neither harm nor benefit), or *pathogens* (of host detriment) [2]–[4].

Current major national research efforts (*i.e.,* the NIH Road Map initiative known as the Human Microbiome Project (HMP)) will enable sequence-based comprehensive characterization of the adult human microbiota and theoretically allow for cataloguing of the microbiota into core guilds, which can be thereafter interrogated for their associations with disease states [1]. Understanding the processes that govern the structure and dynamics of these human microbial communities is essential for gaining a complete understanding of human development and physiology [5]–[9]. However, questions pertaining to how and when diverse microbial communities reside in the host (and how they differ during an individual’s lifetime) are under-explored at a population-wide level. In other words, while we may soon know *what* constitutes the adult human microbial guild, we will neither know *how* it is established nor whether it is dynamic during intervals in reproductive life when the next generation’s microbial community is being established.

Primate fetal development is thought to occur within an intrauterine microbiota-free environment, and yet within a short interval following birth the human microbiome is colonizes and “differentiates” until the adult complement of 90 trillion or so microbiota is achieved [1], [4], [7], [9]. Based on a relative paucity of data, it is proposed that the naïve neonatal microbiome is first established with rupture of the amniotic membranes, with further microbiota being introduced as the fetus traverses the vaginal birth canal. By the time of delivery, the neonate has been exposed to the maternal vaginal microbial ecosystem [9]–[12]. Passage through the vaginal canal is an integral part of this process, as mode of delivery alters the neonatal microbiome [7]–[12]. However, since a comprehensive characterization of the vaginal microbiome signature in pregnancy has not yet been undertaken, conclusions regarding mechanisms of neonatal colonization are likely premature [13]–[17]. Since the infant is exposed to several environmental sources of bacteria in the early neonatal interval (maternal vaginal canal and feces, swallowing and breathing, skin to skin contact, maternal breastmilk, etc.) it is important to discern the relative potential contribution of the maternal vaginal community to the neonate.

Established 16S ribosomal RNA (rRNA) gene sequence-based methodologies have enabled primary cataloguing of the bacterial composition of the human microbiome [18]–[21]. While multiple studies during the past several years have launched the era of human metagenomics, few reports have examined microbiomes outside of the gastrointestinal tract in more than a few individuals [22]–[26], and none have systematically examined the vaginal microbiome throughout pregnancy. Here, we use cultivation-independent, molecular-phylogenetic techniques to characterize the first comparative bacterial assemblages across gestational age strata and in a rigorous clinical study. By interrogating the “healthy” human microbiome in pregnancy and in paralleled comparison with non-pregnancy, we reasoned that the ensuing metagenomic profile would optimally reveal the comparative diversity and richness of microbial species. In this manuscript, we describe our intergroup comparison using rigorous standardized sampling protocols and analyses methodologies in order to provide robust initial evidence that the vaginal microbial gene catalogue uniquely differs in pregnancy, with variance of molecular phylogeny (species richness and diversity) across both subsite and gestational age.

## Results

### Subject Characteristics

Subject characteristics are as outlined in **Table 1**. As anticipated by our use of a parallel protocol design, comparable age, pregravid (or nongravid) BMI, race and ethnicity, and tobacco use were observed among both pregnant and non-pregnant subjects. Although the enrollment percentages by virtue of race and ethnicity were distinct in the two cohorts, these distinctions did not reach statistical significance (p>0.05, both independent samples t-test and ANOVA). As further anticipated among pregnant subjects *per se*, significant variance in medication use with respect to vitamins and antacids were similarly observed. Recalling prospective subject enrollment, the majority of gravidae had uncomplicated pregnancy outcomes: mean gestational age at delivery exceeded 39 weeks and included appropriately grown infants (mean 3265 grams), with 2/24 (8%) of subjects delivering <37 weeks (34 5/7, and 36 6/7 weeks). The cesarean delivery rate was consistent with the regional population (33%). A single fetal comorbidity was observed among the cohort after enrollment (fetal gastroschisis), and a total of 3 subjects manifest 4 comorbidites common to the obstetrical population (**Table 1**). Among the non-pregnant cohort, all subjects were not menstruating at the time of sampling per study protocol, and 58.8% would be anticipated to be anovulatory secondary to use of contraceptive (30 of 51 subjects).

**Table 1:** Subject Characteristics and Pregnancy Outcomes.

*Subject Demographics*	Pregnant n = 24	Non-Pregnant n = 60	
Subject age (mean, years)	31.4 (5.8)	26.9	
BMI (kg/m2) at sampling	30.4 (7.3)	23.9	
BMI (kg/m2) prepregnancy	27.6 (7.6)	NA	
**Ethnicity/Race**			
Hispanic	4 (16%)		11 (18%)
Non-Hispanic	15 (63%)		35 (58%)
Black or African American	3 (13%)		3 (5%)
Other	2 (8%)		11 (18%)
**Tobacco Use**			
Yes	2		4
No	22		56
**Medication (Category)**			
Vitamins or supplements	24		7
Endocrine metabolic agents	1		7
Antacids/H2 antagonists	11		2
GI medication (Antiemetics and stool softeners)	3		2
***Pregnancy Outcomes***			
**Mean gestational age (weeks)**	39 weeks 2 days		
**Preterm birth <37 weeks (rate)**	8% (34 5/7, 36 6/7 weeks)		
**Mean birthweight (grams)**	3265 g		
**Mean Apgar score**			
1 minute	8		
5 minute	9		
**Cesarean delivery (rate)**	33%		
**Vaginal delivery (rate)**	67%		
**Comorbidities**			
(3 subjects with 4 comorbidities)	GDMA1, fetal gastroschisis, mild preeclampsia, severe preeclampsia		

Characteristics of both pregnant (gravidae) and non-pregnant subject cohorts, alongside pregnancy outcomes of the gravid cohort. GDMA1, gestational diabetes mellitus White classification A1 (diet controlled). There were no significant differences with respect to Race/Ethnicity among gravidae and non-pregnant subjects (p>0.05 by independent samples t-test and ANOVA).

### Pregnancy Structures the Vaginal Microbial Community

Other investigators have employed cultivation-independent, molecular phylogenetic approaches to characterizing maternal microbial communities [9]. In order to leverage our data from the HMP, we developed a parallel sampling strategy in our 24 pregnant subjects with equivalent stringent screening criteria [27]. Both subject cohorts were sampled in a uniform and highly consistent manner by a single obstetrician (K.A.) from three distinct vaginal sites (vaginal introitus, posterior fornix, and midvagina); all samples employed in this report were extracted in a single laboratory. With sequencing on the 454 Titanium FLX platform, our approach yielded robust bacterial 16S V5V3 enriched data sets for subsequent analysis (**Table 2**). Because we could not reliably measure nor control for differences in the sampled area or volume, we focused our analyses within these microbial communities on shifts in bacterial community structure and diversity which occur solely by virtue of pregnancy.

**Table 2:** V5V3 Sequence Metrics.

Subject cohort	Number ofSubjects	Number ofSamples	Total Sequences	Average Sequence Length	Average Sequences/Sample
Pregnant	24	68	670,921	498 nt	9,867
Non-pregnant	60	301	1,852,039	491 nt	6,153
Combined	84	369	2,522,960	493 nt	6,837

Sequence metrics for the pregnant (gravidae), non-pregnant, and combined cohorts. Pregnant subject’s samples were of comparable average sequence length, but retained a higher average number of sequence reads per sample.

**Figure 1:**
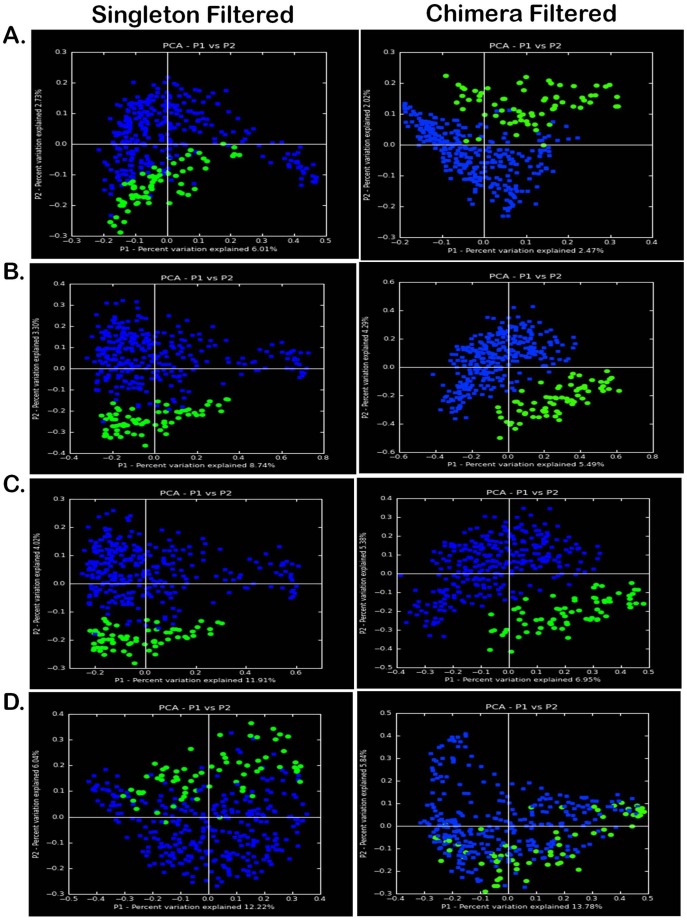
Beta diversity metrics of bacterial 16S rRNA genes reveal distinctly clustered vaginal microbiome communities structured by pregnancy. Datasets were minimally filtered for removal of singletons (left panels) or filtered for chimeras (right panels; QIIME ChimeraSlayer). Beta diversity microbiome community clustering is observed for non-phylogenetic methods ((A) normalized Canberra), binary non-phylogenetic methods ((B) binary Chord, (C) binary Ochiai), and phylogenetic beta diversity metrics ((D) unweighted UniFrac). In each panel, each point corresponds to a vaginal sample from either a pregnant (green) or non-pregnant (blue) subject. The percentage of variation explained by the plotted principal coordinates is indicated on the axes.

As demonstrated in **Figure 1**, microbiome sequence surveys with 16S rRNA pyrosequencing reveal primary structuring by virtue of pregnancy (green versus blue). Given our aim to ultimately describe the vaginal taxa that contribute to a unique community structure in pregnancy, we applied both phylogenetic (UniFrac) and non-phylogenetic (Canberra, Chord, and Ochiai) methods. As a quality filtering step, each dataset was minimally (left plots, **Figure 1**) or modestly (right plots) preprocessed and applied to a flowgram clustering step for removal of chimeric sequences (which occur as a byproduct of the PCR-amplification and pyrosequencing) [28]. This robust analysis approach reveals evident significant community clustering structured predominately by pregnancy regardless of method (phylogenetic or non-phylogenetic) or potential noise for noisy or denoised datasets (**Figure 1**).

**Figure 2:**
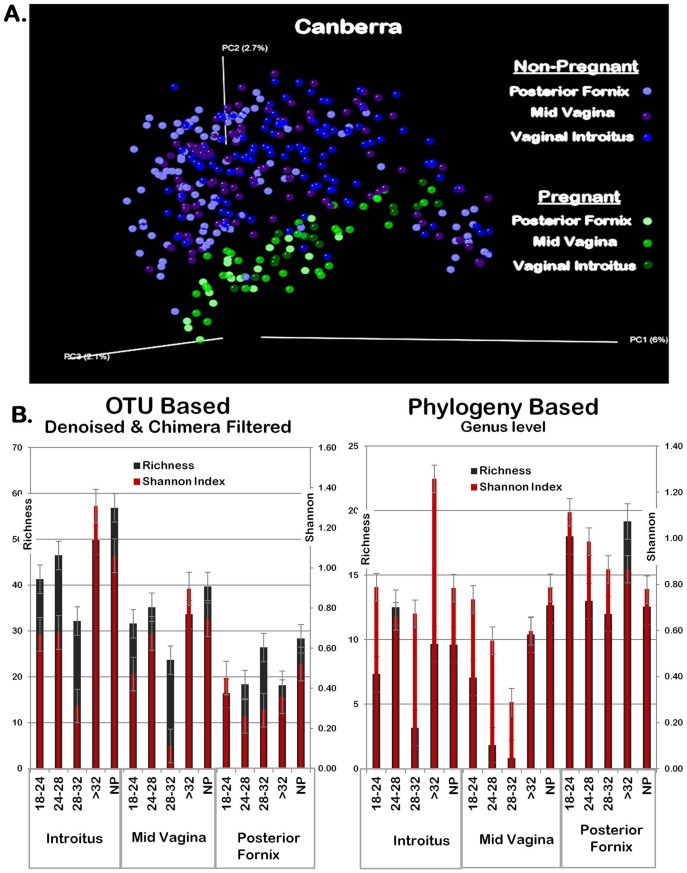
Subclassification of microbial community structure by vaginal subsite and week of gestation. (A) Pregnancy clusters vaginal microbial communities, while site of vaginal sampling minimally contributes to within cluster formation. Canberra beta diversity metric with PCoA plot clustering. Each dots represents one sample from the distinct vaginal subsites (mid vagina, posterior fornix, and vaginal introitus) of individual subjects from pregnant (green shades) and non-pregnant (blue and purple shades). (B) Among gravid subjects, microbial community richness and diversity (Shannon indices) vary by week of gestation and proximity to the uterus. Community richness and Shannon diversity indices by gestational age and vaginal sampling site against normalized abundance values from both OTU and phylogeny based analysis charted by vaginal site (posterior fornix, mid vagina, introitus) and gestational age. Richness - Black; Diversity – Dark Red; Left panel designates OTU based; Right panel designates Phylogeny based. Gestational age interval shown in weeks, or designated as non-pregnant (NP). Error bars denote variance (standard error of the mean, s.e.m.). In each of the gestational age intervals, an equivalent number of gravidae were sampled and compared (n = 6 per strata).

When beta diversity metrics were considered by virtue of the vaginal subsite, pregnancy persisted as the primary arbitrator of community microbial structure (**Figure 2A**). However, when assessing alpha diversity (community richness and Shannon diversity index) with respect to either proximity to the uterus (posterior fornix, which is just posterior to the cervix, versus the vaginal introitus or midvagina) or gestational age (interval in weeks), variable differentiation was observed (**Figure 2B**). When within community (alpha diversity) variance was analyzed with OTU-based methodologic approaches [29]–[33] the vaginal microbiome in pregnancy was equally rich but less diverse than non-pregnant communities (**Figure 2B**, left panel). This result was true in proximity to the cervix and throughout the latter midtrimester as the cusp of fetal viability (24–32 weeks) is reached. However, when taxa were analyzed with phylogenetic applications uniformly projected to the genus level, vaginal introitus and midvaginal communities preterm were both less diverse and less rich than either late preterm (>32 weeks) pregnancy or non-pregnant communities. At the posterior fornix, phylogenetic projections suggested unchanged communities among pregnant and non-pregnant (**Figure 2B**, right panel).

**Figure 3:**
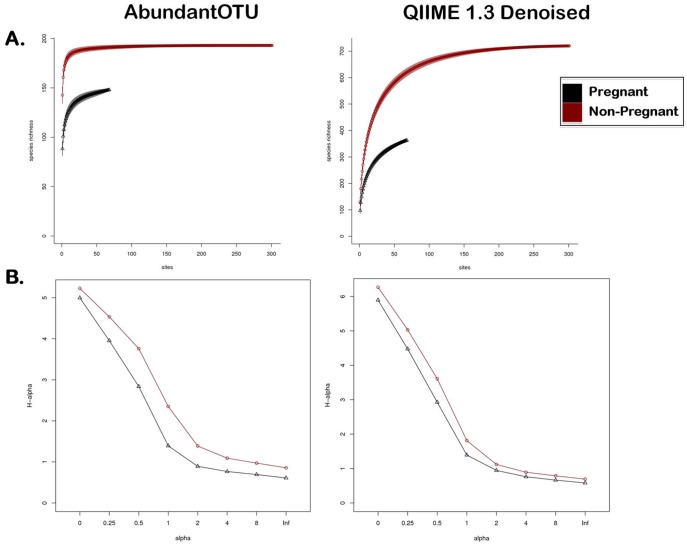
Measures of within community diversity (alpha diversity) at two levels of data filtering. Black lines indicate pregnant cohort, with red lines indicating non-pregnant cohort. Data sets were subjected AbundantOTU (left panels), or denoising and chimera slaying with removal of singletons and chimeras (right panels). (A) Rarefaction alpha diversity metrics note significantly lower richness in the pregnant data set, while (B) Renyi alpha diversity metrics indicates significantly less diversity among pregnant vaginal communities following denoising. Significance is denoted by the absence of curves crossing over at any point following denoising and chimera slaying (right panels).

Given our distinct observations with respect to intracommunity richness and diversity by virtue of taxonomic and phylogenetic approaches, we were concerned with the potential for inflated OTU counts due to sequencing artifacts and/or binning errors. We therefore measured alpha diversity in both pregnant and non-pregnant vaginal communities with AbundantOTU (**Figure 3**, left panels) and QIIME denoising (in addition to chimera and singleton removal) (**Figure 3**, right panels). Although these denoising pipelines did, in fact, differ in their assessments of sequencing noise (and thus resulting OTU counts), both resulted in significant and robust differences among the pregnant (black lines) and non-pregnant (red lines) vaginal microbial communities. They also agreed in that community diversity in pregnancy is both significantly less rich (rarefaction metrics for microbial richness or taxa quantification, **Figure 3A**) [34] and less diverse (Renyi metrics for microbial diversity or number of distinct taxa, **Figure 3B**) [34]–[37]. Taken together, these data are consistent with the notion that community aggregates to genus level projections (**Figure 2B**, right panel) are unchanged in the posterior fornix, but the more robust analysis of genus and sub-genus OTU based projections reveal significantly less diverse and less rich community structure in pregnancy (**Figure 3**).

### Taxa Contributing to the Unique Vaginal Microbiome in Pregnancy

**Figure 4:**
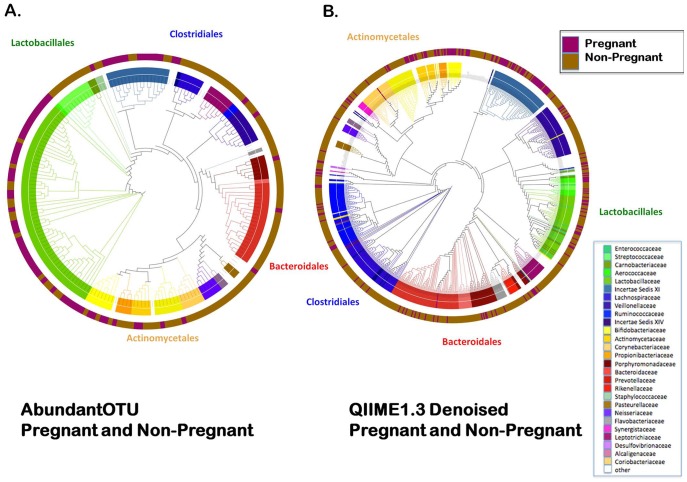
Global phylogenetic trees after AbundantOTU and QIIME denoising. Global phylogenetic trees show the distribution of taxonomy among all the pregnant and non-pregnant subject samples. The internal cluster dendrograms are colored by taxa Family level projections (annotated in figure legend), while the mid-circle is colored by the majority origins of OTUs from pregnant or non-pregnant subject samples (pregnant-magenta; non-pregnant-brown). Outermost circle using text to indicates OTU projection to Order level (Bacteroidales-red, Actinomycetales-yellow, Lactobacillales-green, Clostridiales-blue). OTU tables and representative sequences generated from AbundantOTU (**Figure 4A**) and QIIME denoised (**Figure 4B**) datasets were employed in generating these global phylogenetic trees.

Given the robust evidence that the structure of the vaginal microbiome significantly differs in pregnancy (**Figures 1** and **2**), and further evidence suggesting that the pregnant microbial community is less diverse and rich (**Figure 3**), we next sought to identify which bacterial taxa were contributing to an altered community in pregnancy. OTU tables and representative sequences generated from AbundantOTU (**Figure 4A**) and QIIME denoised (**Figure 4B**) datasets were employed in generating global phylogenetic trees. Additionally, the RDP classifier was used to assign taxonomy and label each element in the OTU table, and the resultant alignment enabled character-based construction of a phylogenetic tree from the filtered NAST alignment using FastTree [38]. Clear family (internal cluster with annotations in figure legend) and order level (middle circle) differences in the pregnancy microbiome are visible in both datasets (**Figure 4**), with an overall predominance of the order Lactobacillales (and *Lactobacillaceae* family), followed by Clostridiales, Bacteroidales, and Actinomycetales. Distinctions between pregnant and non-pregnant communities by unweighted phylogenetic clustering are observed and annotated in the outermost circle (**Figure 4**).

**Figure 5:**
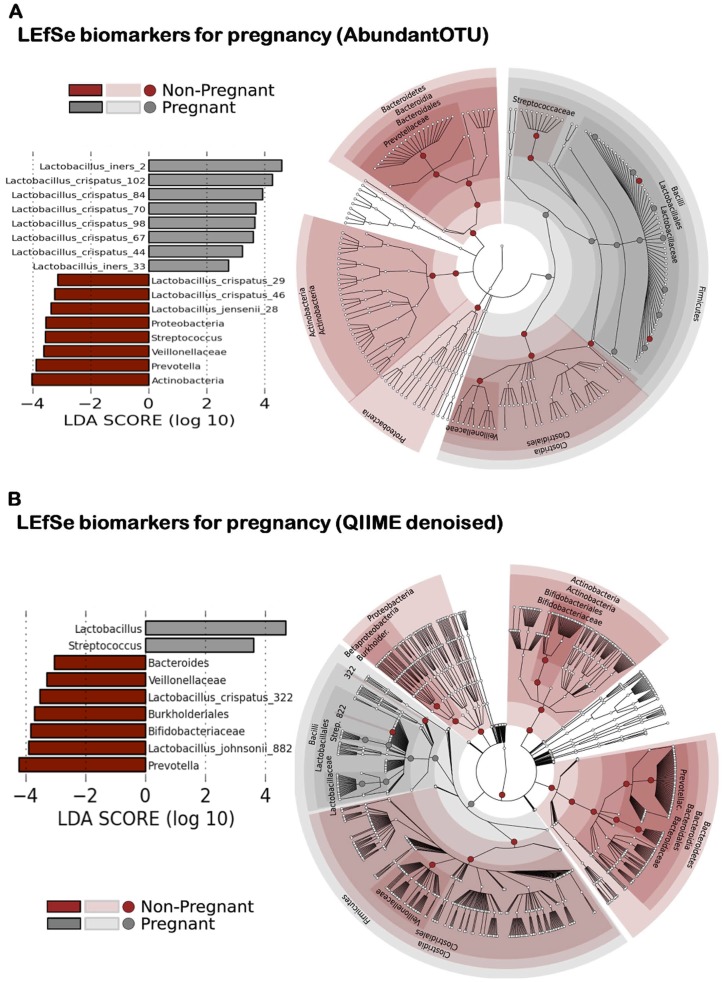
Metagenomics-based discovery of bacterial taxa contributing to differentiation of vaginal communities in pregnancy. Bacterial taxa were selected as significantly differentially abundant between pregnant and non-pregnant communities (regardless of sampling site) by the LDA Effect Size (LEfSe) algorithm, (left panel) sorted by degree of difference (listing only taxonomic leaves) and (right panel) overlayed on a complete taxonomy. Taxa are again reduced in diversity during pregnancy, with several specific *Lactobacillus* OTUs detected uniquely among pregnant individuals. OTU tables and representative sequences generated from AbundantOTU (**Figure 5A**) and QIIME denoised (**Figure 5B**) datasets were employed in LEfSe analyses.

Finally, to identify the specific taxa differentially present or abundant in the vaginal microbiome in pregnancy, we employed two complementary supervised machine learning approaches. The first, the random forest algorithm paired with Boruta feature selection, was validated on classification of subjects by their vaginal microbiome with a pi statistic (improvement over random) exceeding 0.8 at OTU abundance thresholds of 100 or 500 for the AbundantOTU data set (**Figures S1** and **S2**, **Tables S1** and **S2**) and likewise above 0.6 for the QIIME denoised data set (**Figure S1, Tables S1** and **S2**). Pregnancy status was, in fact, the single feature best predicted by the microbiome (as opposed to BMI, vaginal site of sampling, and ethnicity), and sub-sites were not well distinguished (pi statistic of discrimination <0.2 in all cases; **Tables S1** and **S2**, **Figure S3** with binned taxonomy). Using Boruta feature selection during RF analysis, 12 specific taxa were identified as discriminating between pregnant and non-pregnant cohorts at the genus level (at ≥80% bootstrap cutoff, **Figure S1**). In order to identify a taxonomic biomarker with high stringency, we additionally employed the LEfSe method [39], which confirmed the differential abundance of 29 and 27 clades (AbundantOTU and QIIME data, respectively, using default significance and LDA thresholds) at all taxonomic levels between pregnant and non-pregnant microbiomes (**Figure 5**). Supporting our findings in Figures 3 and 4, these comprised primarily specific *Lactobacillus* OTUs and potentially the Bifidobacteriaceae and Streptococcaceae. Our findings suggest that the composition of the microbial community in pregnancy represents a relatively diminished profile of species richness and diversity (**Figures 2** **,** **3** **,** **4** **,** **5**), culminating in the specific reduction of many usual vaginal community members and enrichment for a targeted set of *Lactobacillus* species (**Figure 5**). We present the species-level data in Table S3. Briefly, OTUs generated from AbundantOTU and QIIME were aligned to the Greengenes database with the top OTUs identified by LEfSe (**Figure 5A**). OTU sequences selected by Boruta feature selection are deposited in GenBank and are presented in Figure S2 and Table S3. Top OTUs identified by LEfSe are marked with an asterics. For example, AbundantOTU generated consensus 2/33 aligned to *Lactobacillus iners*, consensus 102/84/70/98/67/44/29/46 to *Lactobacillus crispatus*, and consensus 28 to *Lactobacillus jensenii*. On QIIME, 882 aligned to *Lactobacillus johnsonii*, and 322 to *Lactobacillus crispatus.*

## Discussion

With a robust sampling, sequencing, and analysis approach, we generated the first comprehensive catalogue of the vaginal microbiome in pregnancy across subsite and gestational age. When compared to the non-pregnant vaginal microbiota, the community is uniquely and distinctly structured during pregnancy (**Figure 1** and **2A**), in ways that cannot be attributed to alterations in BMI (**Tables S1** and **S2**), to subject race or ethnicity, nor to readily identifiable clinical confounders. Interrogations of discrete contributors to community diversity revealed that the vaginal microbial community varied in pregnancy by gestational age and proximity to the cervix, but was less diverse and less rich overall (**Figures 2B** and **3**). To our knowledge, this structured molecular study of gravidae is unique in terms of stringency of a parallel clinical approach, sample acquisition from subjects, depth and robustness of analysis, and notable findings. In sum, the vaginal microbiome is distinctly structured by a state of health in most women’s lifetime, *i.e.,* pregnancy.

Others have taken similar but limited approaches to interrogating the vaginal microbiota in pregnancy. Dominguez-Bello utilized 16S 454-generated molecular signatures to generate vaginal profiles in a limited sample set of 9 subjects at term with delivery (including non-laboring and active laboring mothers) from a remote population of Amerindians [9]. In this small sample set, the dominant vaginal taxa varied from mother to mother, also with notable variance in *Lactobacillus* spp. However, these investigations did not include parallel sampling of both non-pregnant and pregnant subjects, nor from multiple vaginal subsites [9]. However, this study was remarkable for its parallel acquisition of neonatal microbial community sampling. As supported by other studies [4]–[12], the infant gut microbiome largely reflects the maternal mode of delivery, although it bears mention that in several studies women were delivered by cesarean for obstetrical indications in active and advanced labor thereby revealing a potential bias by virtue of infant handling in cesarean and vaginal birth and not solely a reflection of fetal descent via the birth canal [9]–[12].

Our study suggests that although human adults have highly differentiated bacterial communities that are relatively stable [3]–[6], [13]–[17], in such prevalent and healthful states as pregnancy the vaginal community in particular shifts naturally in its structure with respect to diversity and richness. Indeed, Ravel *et al* have previously reported that the vaginal microbiome in healthy, reproductive-aged women occupies states dominated by *Lactobacillus iners* and *Lactobacillus crispatus* specifically in association with low vaginal pH [40]. Others speculated [41] that vaginal community changes with pregnancy, but ours is the first direct evidence as such. Indeed, our findings suggest that at least among reproductive aged women, the vaginal microbiome remains a dynamic community in adult reproductive life, and that terminal differentiation does not occur *per se* [41]. Moreover, we observe persistent relative prevalence (but not sole nor absolute predominance) of *Lactobacillus* (**Figure 4**, AbundantOTU). However, across the entirety of our study population, less diversity and richness occurred in measured variance throughout weeks of gestation and in proximity to the uterus (posterior fornix), leading us to speculate on variances within the cusp of preterm viability. Of interest, in subjects closer to term OTU-based projections suggest that the non-pregnant community structure may return to some extent in the latter weeks of gestation. Our study is potentially limited by employing a cross-sectional comparison in gravidae (**Figure 2**), relative to a limited number of non-pregnant subjects with multiple samplings (18/301 specimens represented thrice sampling, see methods). Alternatively, when we compared only first samplings of non-pregnant women to gravidae, we still observed consistent cluster separation (data not shown). The most robust method to formally address the structure shifts in pregnancy would be to employ a longitudinal approach whereby each subject is sampled at ongoing weekly intervals across pregnancy. However, that is outside the scope of this initial study.

We opted to employ a parallel sampling strategy by stringent inclusion and exclusion criteria to the Human Microbiome Project. While this enabled us to make true comparisons to a large, robust, and unparalleled dataset of non-pregnant subjects, it similarly opened the possibility that we were sampling an unperturbed but not “normal” population. However, it bears mention that our outcomes among gravidae were entirely what might be anticipated in a health pregnant population (**Table 2**), and did not differ significantly among subjects. As with all large human cohorts, our study is prone to both alpha error and induced bias. We attempted to minimize error and bias with a single physician performing all subject sampling among both cohorts, and all samples being extracted from primary specimens within a single laboratory utilizing a common and rigorously tested protocol (HMP).

It remains a distinct possibility that our significant observed community clustering with the vaginal microbiome being evidentially structured by pregnancy reflects a secondary trait in our pregnant population, but not gravid condition itself. Of note, we did not exclude gravid subjects by virtue of posterior fornix vaginal pH. In contrast, in the non-gravid HMP cohort subjects were excluded at the time of screening if the posterior fornix pH exceeded (see Methods). Given that <10% of screen failures (and <4% of the entire potential cohort) met such pH criteria for exclusion, we feel that this is an exceedingly unlikely potential confounder or bias. However, it cannot be formally excluded as such. To this end, in the recent publication of Ravel *et al.,* [40], the authors reported that while the pH and Nugent scores of each community demonstrated strong correlation between high pH and high Nugent scores and the highest pH values were associated with community states not dominated by species of Lactobacillus. It is of importance to note that these investigators employed self-sampling in their study. Nevertheless, the investigators also reported that elevated pH and high Nugent scores were observed in some communities with high proportions of lactobacillus species and that this was most true in communities which contained decreasing proportions of *L. iners*. However, this was not universally true, leading the authors to summize that these metrics cannot be predicted with absolute certainty solely on the basis of the proportion of Lactobacillus in a community [40]. We concur with these investigators summary statements, and note that when comparing our population of pregnant subjects to the non-pregnant HMP cohort we observed community discrimination by virtue of Lactobacillus species, namely *L. iners*, *L. crispatus*, *L. jensenii* and *L. johnsonii*. If our community distinctions were the result of an incidental inclusion of pregnanc subjects with a posterior fornix pH >4.5, then we would anticipate potentially seeing a decreasing proportion L. iners in the community structure. However, the opposite holds true (**Figure 5**) making confounding by version of pH unlikely.

As the number and robustness of computational approaches to analysis of metagenomics data increases, investigators are faced with distinct methodologic approaches to analyzing community profiles. In any emerging field of study, optimal measures of data analysis are not evident to investigators at the forefront and different methodologic approaches may yield variance in significance of findings [28], [42]–[46]. With this in mind, we employed a diverse and robust set of bioinformatic tools in analysis of our datasets (see Methods). For community cluster distinction (beta metrics), we analyzed taxa by nonphylogenetic and phylogenetic methods. Regardless of distance metric or phylogenetic analysis, the vaginal microbiome distinctly clustered by virtue of pregnancy (**Figures 1** **,** **2A** **,** and **4**). As AbundantOTU uses a consensus alignment algorithm, thus tending to concentrate on OTUs of greater abundance. Detection of rare species is difficult to differentiate from sequencing error. QIIME denoiser preempts this difficulty with a pre-filter to reduce the needs of all-on-all comparison; each additional unclustered read is compared to the most abundant clusters to discern sequencing error probability from detection of rare bacterial species retained in the OTU table. Regardless of methodology, our results from AbundantOTU and QIIME denoising are strikingly similar in terms of differentiating OTU identified (**Figure 4**). This finding is further evident at the species level, as detailed in **Figure 5**.

Similarly, for measurement of within community diversity (alpha) we employed variations in data filtering ranging from minimal removal with scant trimmed reads to well-described modest “denoising” and slaying of chimeras. We persistently observed less richness and diversity in pregnant communities when compared with parallel non-pregnant subject cohorts, regardless of computational pipeline, tool employed, or means of data projection (**Figures 2B** and **3**). The limited results analyses at the genus level, and the number of significantly differential OTUs (**Figure 5**), both suggest that it is subgenus taxa that most strongly contribute to observed alterations in community structure in pregnancy. This was supported by two complementary rigorous denoising approaches to our dataset. AbundantOTU and QIIME. The former resulted in diminished OTU estimates (868 versus 1,121 OTUs), but both agreed in the predominance of lactobacilli (**Figure 4**) irrespective of gravid condition and clades differential during pregnancy (**Figure 5**). With QIIME denoising, a broader set of taxon differences and less absolute predominance of *Lactobacillus* could be observed. While each denoising pipeline has its own strengths and limitations, one undeniable observation persists: the vaginal microbiome community is structured by pregnancy and varies with respect to richness, diversity, and specific microbial members.

Employing such robust analyses methods, we were able to detect species which are discriminately and specifically relative enriched in pregnancy (albeit in the face of overall diminished community richness and diversity). These include *Lactobacillus iners*, *Lactobacillus crispatus*, *Lactobacillus jensenii* and *Lactobacillus johnsonii*. Although it is outside the scope of this initial manuscript to delve deeply into the species differentiation and clinical implications, these findings are of probable biologic significance nevertheless. For example, *L. johnsonii* encodes enzymes and transporters essential for the release bile salt hydrolase and is primarily found in the upper GI tract [47]. In addition, the capacity for production of bacteriocins is a broad trait of the lactic acid bacteria and *L. johnsonii* production of Lactacin F both limits other lactobacillus as well as Enterococcus species in the GI tract. It’s notable increased dominance in the vagina in pregnancy may be important for establishing the neonatal upper GI microbiota upon delivery, or preserving the integrity of the community to reduce risk of ascending infection or preterm birth.

The vaginal microbiome signature in pregnancy is thus distinct from non-pregnant, and this distinction comprises both from lesser diversity and, to a lesser degree, from the absence and occasionally presence of unique taxa. Our reporting by gestational age and vaginal subsite now lays the foundations for further interrogations into microbial variance, including such presumed pathogen-related perinatal morbidities as preterm birth. Moreover, it lends to the growing understanding of the remarkable dynamic nature of our metagenome and its role in vertical transmission of the microbiota through subsequent generations.

## Materials and Methods

### Subjects

The intent of this study was to compare the vaginal microbiome in pregnancy from healthy individuals whose core microbiomes were likely to be minimally perturbed by virtue of infectious comorbidities and exogenous exposures. We took advantage of our role as clinical investigators with the Human Microbiome Project (K.A., J.P., and J.V., Baylor College of Medicine) [27] and employed parallel recruitment of non-gravid (non-pregnant) and gravid subjects using rigorous standardized sampling protocols. Subjects were recruited from the general population with general media and institutional advertisements, in addition to institutional study enrollment web sites and approach during previously scheduled clinical appointments. An initial telephone query included a general health questionnaire to screen interested individuals who would be evaluated by a list of inclusion/exclusion criteria including a pre-pregnancy or current body mass index range of 18–35, history of cancer, compromised immune status, history of specified chronic diseases, or medication exposure within the last six months (*e.g.,* antibiotics, corticosteroids, cytokines, large doses of probiotics, etc.). Major dietary changes and history of moderate-high alcohol intake excluded individuals. Medications and dietary components that potentially might alter the human microbiome intentionally such as antibiotics and probiotics served as additional important exclusion criteria. Females were required to have a regular 21–35 day menstrual cycle or a history of regular cycles prior to beginning contraception or at the time of conception, and the use of specific contraceptives such as the combination hormone vaginal ring was exclusionary in non-gravid subjects. For gravidae, inclusion criteria additionally detailed best obstetrical dating for gestational age, defined as reliable last menstrual period consistent with <12 week sonogram or <10 week sonographic dating with unreliable last menstrual period. At the time of enrollment, all gravid subjects had presumed normal singleton gestations and were without known maternal comorbidities (such as type II DM, gestational diabetes type A2, hypertensive disorders, or chronic medical conditions). However, given the prospective nature of the study with early second trimester enrollment, some subjects developed these comorbidities (**Table 1**). Individuals who remained interested in the study and met study criteria were invited for a screening visit in the research clinic (non-gravid), or approached at their next scheduled clinic visit (gravid).

At the screening visit, the study and associated risks were explained and subjects documented informed consent by signing a study consent form. Investigators determined final study eligibility by taking a medical history and performing a review of systems, documenting concomitant medications, measuring vital signs, collecting urine for pregnancy testing (non-gravid) and either obtaining blood for serum HIV, HBV and HCV testing or reviewing previously obtained serologies (all required to be negative). In addition, all subjects underwent targeted physical exams with attention to body site-specific exclusion criteria (oral cavity, skin and nasal cavity), and among non-gravid subjects measuring vaginal pH in females (pH>4.5 at posterior fornix was exclusionary). Gravid subjects did not undergo vaginal pH measurements as per study protocol limitations but were queried as to signs and symptoms of bacterial vaginosis and excluded if reported. Individuals who passed all screening criteria were eligible for enrollment and were sampled the first time within 30 days of the screening visit (non-gravid), or at the time of the prenatal visit (gravid). Although exclusion of non-gravidae for posterior fornix pH>4.5 at the time of screening was exceedingly rare (notably with only 22 of 254 screen failures in the HMP occurring by virtue of vaginal irritation or posterior fornix pH>4.5; 554 potential subjects were screened to ultimately enroll 300 in total for the HMP [http://www.hmpdacc.org/micro_analysis/microbiome_sampling.php]), it remains a formal possibility that our cohort comparison may be biased as posterior fornix pH was not an exclusionary criteria in our pregnant cohort.

For the non-gravid (non-pregnant) comparison cohort, of the 150 females from the HMP JumpStart initiative [18], [19] all individuals were designated for study enrollment *a priori*. Enrollment criteria were established at approximately 20% minority (racial and ethnic) subjects. While in the index study protocol, two ethnically, racially, and socieconomically diverse U.S. cities (Houston, Texas and St. Louis, Missouri) served as geographically distinct study sites for clinical sampling for this comparison. However, only subjects sampled at Baylor College of Medicine were considered in the comparison cohort. This served to limit potential additional variables, including personnel doing the sampling (all gravid and non-gravid subjects were sampled by one investigator [K.A.]), site of microbial DNA extraction, and regional variability. To assure compliance of screening and sampling measures, a common set of human study protocols and consent forms were created, reviewed independently and subsequently approved at Baylor College of Medicine. An age range of 18–40 years was established to minimize variability due to childhood growth and development, aging, and hormonal influences during adolescence and menopause. Each of the enrolled non-gravid subjects agreed to primary and repeat (second) sampling so as many individuals as possible were sampled twice. A subset of non-gravid subjects was designated to undergo three samplings at each body site, at *a priori* designated intervals.

### Subject Compliance/Protection and Informed Consent

Specimen collection procedures involved minimal physical risk to subjects. As defined in 45 US Code of Federal Regulations (CFR) 46.102 (i), “Minimal risk” infers that the “probability and magnitude of harm or discomfort anticipated in the research are not greater in and of themselves than those ordinarily encountered in daily life or during the performance of routine physical or psychological examinations or tests.” Potential risks of participation as discussed with subjects included those associated with biologic sample collection and repository, and the unintentional release of protected health information; the protocol and informed consent form described precautions taken to reduce these risks. The study sites developed informed consent documents using NHGRI guidelines for genomics studies, which address the ethical, legal and social implications of such research. The Institutional Review Board reviewed and approved the protocol, informed consent and other study documents. Additional protections for subjects included Certificates of Confidentiality intended to protect against the forced disclosure of identifiable research data, coding of genomic and metagenomic specimens and sequence data, and use of controlled access databases for medical data and human genome sequence data. As part of the consent process, we informed subjects about data protection, including coding, use of controlled-access databases for human genomic data and controlled-access repositories for extracted metagenomic nucleic acids. If a subject withdrew consent after providing specimens, remaining specimens and extracted nucleic acids were to be destroyed; however, any metagenomic sequence data that was already published in open access databases could not be retracted.

### Clinical Metadata Collection

EMMES and the Data Analysis and Coordination Center for the Human Microbiome Project (www.hmpdacc.org) established an internet data entry system for investigators to enter coded non-gravid subject information; gravid subject information was entered and maintained on a local database with secured protection but not released to the DACC. Clinical data elements included gender, race, ethnicity, age, place of birth, occupation, body mass index, vital signs, vaginal pH (non-gravid), date of last menstrual period and first sonogram, tobacco use, and both dental and health insurance status. A medical history, comprehensive obstetrical data, and targeted physical examination findings alongside medication history were also recorded for each subject.

### Specialized Reagents and Instrumentation for Human Sampling

The Catch-All swab from Epicentre Technologies was selected as the swab of choice for sampling based on preliminary evaluations done in conjunction with HMP. The Catch-All swab was used for collection of all vaginal samples. Prior to vaginal sampling in non-gravid subjects, a digital display pH meter with accuracy to >0.01 was employed for precision measure of vaginal pH (Waterproof BigDisplay pH Spear, Oakton pH meter, Vernon Hills, IL.).

### Subject Sampling

Inclusion criteria included stringent requirements as otherwise detailed [27]. Healthy, young adults (ages 18–40) who retained the ability to provide informed consent and were willing and available to provide samples during the study interval were targeted for enrollment. In non-gravid subjects, specimen types included anterior nares, oral cavity (9 samples), peripheral blood, skin (4 samples), stool, and vaginal (3 samples per female) samples [27]; in gravidae, only vaginal samples at the posterior fornix, midvagina, and vaginal introitus were collected. All subjects were required to have a minimum of 24 teeth with no more than 8 missing teeth. Three vaginal specimens were collected in a systematic and uniform fashion from the vaginal introitus, the posterior fornix, and the midpoint of the vagina. All specimens were collected using sterile Catch-All™ specimen collection swabs by applying the swab to a single site, swirling it 6 times, and then withdrawing from the site without contamination. The protocol for collection was as follows: in non-gravid subjects, the pH was measured at the vaginal introitus (Oakton pH meter, Vernon Hills, IL) and among both cohorts the vaginal introitus specimen was collected first. A clear, small or medium Pederson speculum (manufacturer) was thereafter introduced in the absence of lubrication, and turned to an approximate 45 degree angle to enable placement of the Oakton pH meter at the posterior fornix for determination of posterior fornix pH (non-gravid subjects). In both gravid and non-gravid, the posterior fornix sample was collected, then the speculum was slowly withdrawn to the midpoint of the vagina and the midpoint specimen was obtained. Subjects were not menstruating on the days of specimen collection and were abstaining from sexual intercourse, douching, tampon usage or vaginal creams for the preceding 48 hours.

### Specimen Processing

In this study protocol, vaginal specimens were coded, stored and processed for nucleic acid extraction at a single laboratory (J.P.) using a common protocol to reduce variability between samples. Genomic DNA was isolated on standard protocol with PowerSoil DNA Isolation Kit (MoBio) per HMP modifications (http://www.hmpdacc.org/doc/HMP_MDG_454_16S_Protocol_V4_2_102109.pdf).

### 16S Metagenomic 454 Sequencing Data Generation

Extracted DNA samples were used for 16S rRNA sequence-based survey. High Fidelity PCR reactions were performed in 96 well plates. 16 µL of master mix composed of 13.85 µL RNAse/DNAse free water, 2 µL 10X AccuPrime PCR Buffer II, and 0.15 µL AccuPrime™ Taq DNA Polymerase (Invitrogen) were mixed into individual wells in the 96 well reaction plate. The covered plate was spun and centrifuged at 2000 rpm to collect sample at the bottom of the wells. For the initial reaction, a 2 µL sample of DNA was added to the reaction wells. Barcoded primers (2 µM) from the primer plate were added to corresponding wells in the 96 well PCR plate. Two different PCRs were set up separately with a set of barcoded primers targeting the V3V1 region and V3V5 regions; this analysis examined V3V5 regions. The V3V5 regions of the 16S rRNA gene were amplified by PCR using bar-coded universal primers 354F and 926R (V3-V5) containing the A and B sequencing adaptors (454 Life Sciences, Branford, CT) obtained from Invitrogen. Primer sequences are as follows:B-354F(5′-cctatcccctgtgtgccttggcagtctcaGCCTACGGGAGGCAGCAG-3′; B adaptor in lowercase letters); A-926R (5′ccatctcatccctgcgtgtctccgactcagNNNNNCCGTCAATTCMTTTRAGT; A adaptor in lowercase letters, and N represents a bar code that is unique for each sample). Cycling conditions were 95°C for 2 min, followed by 30 cycles at 94°C for 20 s, 50°C for V3V5 primer sets. PCR products were cleaned using AmPure Beads Agencourt (Beckman Coulter, Beverly, MA) using 1.8× volume beads. Beads were eluted with 25 µL 1× low TE, pH 8.0 and transferred to a new 96-well plate. PCR products were quantified using Quant-IT dsDNA high sensitivity assay (Invitrogen) according to the manufacturer’s specifications. All samples were diluted according to the sample that had the lowest concentration. Equal volumes of each (5–10 µL) sample were pooled and then concentrated using MinElute columns (Qiagen, Valencia, CA). DNA pool emulsion PCR amplification and 454 sequencing were performed at the BCM Human Genome Sequencing Center (HGSC) in Houston, TX. Sequencing was performed using the 454/Roche B sequencing primer kit in the Roche Genome Sequencer GS-FLX Titanium platform. Samples were combined in a single region of the picotiter plate such that approximately 20,000 to 40,000 sequences were obtained from each group with each primer set. Samples were isolated and quality-filtered from each multiplexed Standard Flowgram Format (SFF) file.

In the gravid sample cohort, three subjects did not have valid sequences from a total of 4 body sites (subject 14 did not have valid sequences from midvagina nor posterior fornix, and subjects 6 and 9 did not have from the midvagina and posterior fornix, respectively). In the non-pregnant cohort, 29 subjects were sequenced for all three vaginal subsites 1 time (29 of 301 samples), 21 subjects for all three subsites twice (42 of 301 samples), 6 subjects thrice (18 of 301 samples), and 2 subjects for all three subsites four times (8 of 301 samples).

### Denoising Data Sets

As a quality filtering step, each sample was preprocessed to remove sequences with length less than 200 nucleotides and sequences with minimum average quality less than 20. If they could be identified, reverse primers were also removed from sequences. In a first pass denoising, singletons and chimeric sequences (which occur as a byproduct of the PCR-amplification) were identified by ChimeraSlayer [28] and removed from the representative sequence file generated by the output of the minimal filtering pipeline and subsequent OTU table.

For second pass denoising, two programs were employed: AbundantOTU [29] and the QIIME denoising pipeline. AbundantOTU is a robust and fast OTU picking approach, which uses a consensus alignment algorithm to infer consensus sequences from full-length 16S pyrosequences. Since it relies on sequence redundancy, sequencing errors will have less effect on the OTU picking process compared with other clustering based methods. Rare species, which tend to cause inflated species diversity estimations [30], are not included. In our case, 87% of all input sequences were assigned to 194 consensus sequences. For QIIME denoising, the sequences from all samples were processed through QIIME’s 454 dataset denoising pipeline (version 1.3), whereby the sff files from 301 non-pregnant samples and the sff file from the 68 pregnant samples were grouped into one complete dataset for denoising [31], resulting in the production of an OTU table for all 369 samples in a single run on a local cluster utilizing 75 processing cores [32], [42], [43]. Chimeric sequences in the representative sequence sets picked from denoised fasta files were detected by ChimeraSlayer and subsequently removed from the OTU table. In order to account for artifacts that may arise from multiple sequencing runs and separate denoising runs, all samples were denoised together in all alpha diversity, supervised and unsupervised learning, and feature selection pipelines.

### 16S Metagenomic 454 Sequencing Analyses

#### RDP pipeline

Unique reads were classified with the MSU RDP classifier v2.2 [44], maintained at the Ribosomal Database Project (RDP 10 database, version 6), and normalized data were produced from the relative abundance of taxa present in each sample based on a naïve Bayesian classifier [28], [43]–[45]. Output sequences were classified as domain, phylum, family and genus, depending on the depth of reliable classifier assignments. Combined sample reports included the total counts and normalized (relative abundance) data.

#### Creating input data

QIIME was utilized to produce OTU tables from the quality-filtered sequences as outlined above [28]–[32], [42]–[44]. The generated OTU tables combined with the clinical metadata comprised the data matrix used as input for alpha diversity (biodiversity within a group of samples), beta diversity (biodiversity between groups of samples) [3], and machine learning pipelines (randomForest). Quality filtered sequences were analyzed using three standard microbiome analysis techniques: operational taxonomic unit (OTU) generation, phylogenetic tree construction, and taxonomic binning of classified sequences.

#### Alpha diversity

A custom in-house pipeline has been written to calculate alpha diversity measurements and plots using R packages [46]: BiodiversityR [48] and vegan [49]. This pipeline was designed to utilize the output of the OTU picking step (OTU table and meta data file) as the only necessary input. Analyzing the biodiversity within a group of samples provides insight into the differences in species richness, evenness, abundance; we employed the methods of Chao1 [35] and Shannon [36] as we were analyzing groups of samples. Plots were generated and exported for species richness, rank-abundance, and Renyi’s diversity indices [34] whereby species richness plots depict the number of species (OTUs) on the y-axis and the number of sites (samples) on the x-axis such that rank-abundance curves rank and list the most abundant OTUs from left to right on the x-axis and top to bottom on the y-axis. The width of the curve on the on the horizontal axis of a species richness plot serves as an indicator of richness: a wide curve indicates higher species richness and a narrow curve indicates the opposite. The shape of the rank-abundance curve is an indicator of species evenness: a horizontal curve indicates a completely evenly distributed system, whereas a steep curve indicates a less even distribution of species [48]. Rank abundance has been demonstrated to successfully show the extent that tag pyrosequencing illuminates the rare biosphere of the human gut [37]. Based on prior evidence, we assumed that Renyi diversity profiles are helpful in analyzing the differences in diversity and evenness between multiple subsets of samples [34], [37], [50]. If the curves from one sample set contain greater y-axis values, then it can be concluded that this sample set has greater diversity. However, if the two curves intersect at any point, the sample sets are said to be non-comparable, which may reflect important ecological processes [51].

#### Beta diversity

Beta diversity analysis incorporated 9 binary non-phylogenetic (binary chi-square, binary chord, binary Euclidean, binary Hamming, binary Jaccard, binary Lennon, binary Ochiai, binary Pearson, and binary Sörensen-Dice), 14 non-phylogenetic (Bray-Curtis, Canberra, Chi-square, Chord, Euclidean, Gower, Hellinger, Kulczynski, Manhattan, Morisita-Horn, Pearson, Soergel, Spearman rank, and species profile), and 6 phylogenetic (UniFrac G metric, UniFrac full tree, unweighted UniFrac, unweighted UniFrac full tree, weighted normalized UniFrac, and weighted UniFrac) beta diversity metrics [51]–[54]. Each beta diversity distance metric calculated was systematically displayed for the top 3 principal coordinates (PCoA (Principal Coordinates Analysis)) for both normalized and non-normalized OTU tables in both 2D and 3D formats for further analysis [55].

#### Phylogenetic analysis

Phylogenetic trees were produced as necessary input for the phylogenetic beta diversity metrics (*i.e.* UniFrac) in order to determine whether derived microbiome communities were significantly different as an estimate of the degree of divergence between different representative sequences [53]–[55]. Phylogenetic trees were produced in Newick format employing interactive tree of life (iTOL) interface [56].

#### Machine learning (randomForest modeling with Boruta feature selection and LEfSe)

In order to supplement the data obtained from the taxonomically binned reports, alpha diversity, beta diversity, and phylogenetic data sets, a machine learning pipeline was written to study the patterns that can be detected within the sub-groups of various microbiomes [33], [38], [39], [57]–[63]. Machine learning algorithms are useful in determining the strength of meta data clusters (bagging, binning, etc.) as well as listing the most important variables involved in discriminating two groups of samples (feature selection). The algorithm randomForest enabled classification of groups of samples by constructing a classification tree, randomly sampling the predictors, choosing the best splitting variables, and predicting new data by combining the predictions from all trees in order to estimate the error rate [“out-of-bag” (OOB)] and list the highest performing variables. We secondarily applied the R package Boruta to explicitly perform feature selection [33], [39], [58]–[60]. Linear discriminate analysis effect size (LEfSe) is a novel method developed to support for high dimensional class comparisons in metagenomics analysis [61]. LEfSe combines the standard tests for statistical significance (Kruskal-Wallis test and pairwise Wilcoxon test) with linear discriminate analysis for feature selection. In addition to detecting significant features, it also ranks features by effect size, which put features explain most of the biological difference at top. Pregnant and Non-pregnant were indicated as two classes with no subclass indicated. Alpha value for the factorial Kruskal-Wallis test is 0.05. Threshold on the logarithmic LDA score for discriminative features is 2.0.

## Supporting Information

Figure S1 **Accuracy of phenotypic predictions from microbiome composition by randomForest.** OTU abundances using increasingly many minimal row contributions were used by RF machine learning to predict clinical metadata including pregnancy status, vaginal sampling subsite, subject body mass index, and race/ethnicity. Pregnancy was extremely well predicted by OTU features (Scott’s pi >0.8, as compared to a random baseline of 0), exceeding the (still high) accuracy of other metadata predictions.
(TIF)

Figure S2 **Supervised (machine) learning with definition by randomForest and confirmation by Boruta feature selection enables visualization of bacterial taxa contributing to clustering of vaginal communities in pregnancy**. Bacterial taxa (leftmost column) were defined by randomForest (Table S1) and confirmed by Boruta feature selection. Taxa are sorted first by Mann-Whitney U score, followed by the largest disparity in medians for each group. Taxa represent the lowest taxonomic depth (Genus) that are labeled by RDP Classifier (at ≥80% bootstrap cut off). Boxes represent the first quartile, median, and third quartile of the distribution of OTUs for each sample group. Empty circles represent outliers that are 1.5-fold greater than the respective interquartile ranges.
(TIF)

Figure S3 **LEfSe analysis of binned taxonomy at discrete vaginal subsites.** Bacterial taxa were selected as significantly differentially abundant between pregnant and non-pregnant communities by virtue of discrete sampling site and displayed by LDA Effect Size (LEfSe) algorithm. Taxa level projections are defined by pregnancy at each subsite, with specific *Lactobacillus* species detected consistently among pregnant individuals.
(TIF)

Table S1 Estimated error rate of the randomForest simulation by virtue of potentially contributable clinical metadata (pregnancy, BMI, vaginal sampling site, and ethnicity) following abundant OTU pipeline for denoising of dataset. Top row header: Minimal row contribution cut off sum for each OTU to determine the best performing data set (*i.e.,* contains the most discriminative features with least amount of noise). When describing estimated error rate per minimal row contribution, pregnancy was retained as the only significant clinical metadata category in the model simulation that had an acceptable level of estimated error (<10%, in bold face type).
(DOC)

Table S2 Estimated error rate of the randomForest simulation by virtue of potentially contributable clinical meta data (pregnancy, BMI, vaginal sampling site, and ethnicity) following QIIME pipeline for denoising of dataset. Top row header: Minimal row contribution cut off sum for each OTU to determine the best performing data set (*i.e.,* contains the most discriminative features with least amount of noise). When describing estimated error rate per minimal row contribution, pregnancy was retained as the only significant clinical metadata category in the model simulation that had an acceptable level of estimated error (<10%, in bold face type).
(DOC)

Table S3 The OTUs selected by Boruta feature selection are assigned to the species level by realigned to Greengenes database and BLAST with NCBI microbial database. Top OTUs identified by LEfSe are marked with an asterics.
(DOC)

## References

[1] Turnbaugh PJ, Ley RE, Hamady M, Fraser-Liggett CM, Knight R (2007). The Human Microbiome Project.. Nature.

[2] Qin L, Li R, Raes J, Arumugam M, Burgdorf KS (2010). A human gut microbial gene catalogue established by metagenomic sequencing.. Nature.

[3] Costello EK, Lauber CL, Hamady M, Fierer N, Gordon JI (2009). Bacterial community variation in human body habitats across space and time.. Science.

[4] Ley RE, Peterson DA, Gordon JI (2006). Ecological and evolutionary forces shaping microbial diversity in the human intestine.. Cell.

[5] Ley RE, Hamady M, Lozupone C, Turnbaugh PJ, Ramey RR (2008). Evolution of mammals and their gut microbes.. Science.

[6] Ley RE, Lozupone CA, Hamady M, Knight R, Gordon JI (2008). Worlds within worlds: evolution of the vertebrate gut microbiota.. Nat Rev Microbiol.

[7] Penders J, Thijs C, Vink C, Stelma FF, Snijders B (2006). Factors influencing the composition of the intestinal microbiota in early infancy.. Pediatrics.

[8] Turnbaugh PJ, Hamady M, Yatsunenko T, Cantarel BL, Duncan A (2009). A core gut microbiome in obese and lean twins.. Nature.

[9] Dominguez-Bello MG, Costello EK, Contreras M, Magris M, Hidalgo G (2010). Delivery mode shapes the acquisition and structure of the initial microbiota across multiple body habitats in newborns.. Proc Natl Acad Sci.

[10] Favier C, de Vos W, Akkermans A (2003). Development of bacterial and bifidobacterial communities in feces of newborn babies.. Anaerobe.

[11] Mackie RI, Sghir A, Gaskins HR (1999). Developmental microbial ecology of the neonatal gastrointestinal tract.. Am J Clin Nutr.

[12] Gronlund MM, Lehtonen OP, Eerola E, Kero P (1999). Fecal microflora in healthy infants born by different methods of delivery: Permanent changes in intestinal flora after cesarean delivery.. J Pediatr Gastr Nutr.

[13] Forney LJ, Foster JA, Ledger W (2006). The vaginal flora of healthy women is not always dominated by Lactobacillus species.. J Infect Dis 194: 1468–1469; author reply 1469–1470.

[14] Brown CJ, Wong M, Davis CC, Kanti A, Zhou X (2007). Preliminary characterization of the normal microbiota of the human vulva using cultivation-independent methods.. J Med Microbiol.

[15] Forney LJ, Gajer P, Williams CJ, Schneider GM, Koenig SS (2010). Comparison of self-collected and physician-collected vaginal swabs for microbiome analysis.. J Clin Microbiol.

[16] Kim TK, Thomas SM, Ho M, Sharma S, Reich CI (2009). Heterogeneity of vaginal microbial communities within individuals.. J Clin Microbiol.

[17] Xhou X, Brotman RM, Gajer P, Abdo Z, Schuette U (2010). Recent Advances in Understanding the Microbiology of the Female Reproductive Tract and the Causes of Premature Birth.. Infect Dis Obstet Gynecol.

[18] Zoetendal EG, Akkermans AD, DeVos WM (1998). Temperature gradient gel electrophoresis analysis of 16SrFNA from human fecal cells reveals stable and host-specific communities of active bacteria.. Appl Environ Microbiol.

[19] Eckburg PB, Bik EM, Bernstein CN, Purdom E, Dethlefsen L (2005). Diversity of the human intestinal microbial flora.. Science.

[20] Favier CF, Vaughan EE, DeVos WM, Akkermans AD (2002). Molecular monitoring of succession of bacterial communities in human neonates.. Appl Environ Microbiol.

[21] Hooper LV, Wong MH, Thelin A, Hansson L, Falk PG (2001). Molecular analysis of commensal host-microbial relationships in the intestine.. Science.

[22] Reinhardt C, Reigstad CS, Backhed F (2009). Intestinal microbiota during infancy and its implications for obesity.. J Pediatr Gastr Nutr.

[23] Penders J, Thijs C, Vink C, Stelma FF, Snijders B (2006). Factors influencing the composition of the intestinal microbiota in early infancy.. Pediatrics.

[24] Qin J, Li R, Raes J, Arumugam M, Burgdorf KS (2010). A human gut microbial gene catalogue established by metagenomic sequencing.. Nature.

[25] Gill SR, Pop M, Deboy RT, Eckburg PB, Turnbaugh PJ (2006). Metagenomic analysis of the human distal gut microbiome.. Science.

[26] Kurokawa K, Itoh T, Kuwahara T, Oshima K, Toh H (2006). Comparative metagenomics revealed commonly enriched gene set in human gut microbiomes.. DNA Res.

[27] Aagaard KJ (2011). A comprehensive strategy for sampling the human microbiome.. Sci Transl Med.

[28] Haas B, Gevers D, Earl AM, Feldgarden M, Ward DV (2011). Chimeric 16S rRNA sequence formation and detection in Sanger and 454-pyrosequenced PCR amplicons.. Genome Res.

[29] Ye Y (2010). Identification and quantification of abundant species from pyrosequences of 16S rRNA by consensus alignment.. Proceedings (IEEE Int Conf Bioinformatics Biomed).

[30] Quince C, Lanzen A, Curtis TP, Davenport RJ, Hall N (2009). Accurate determination of microbial diversity from 454 pyrosequencing data.. Nat Methods.

[31] Reeder J, Knight R (2010). Rapidly denoising pyrosequencing amplicon reads by exploiting rank-abundance distributions.. Nat Methods.

[32] Caporaso JG, Kuczynski J, Stombaugh J, Bittinger K, Bushman FD (2010). QIIME allows analysis of high-throughput community sequencing data.. Nat Methods.

[33] Knights D, Costello EK, Knight R (2011). Supervised classification of human microbiota.. FEMS Microbiol Rev.

[34] Renyi A, Neyman J (1961). On measures of entropy and information..

[35] Chao A (1984). Nonparametric estimation of the number of classes in a population.. Scand J Stat.

[36] Shannon CE (1948). A mathematical theory of communication.. Bell System Technical Journal 27: 379–423 and 623–656.

[37] Dethlefsen L, Huse S, Sogin ML, Relman DA (2008). The pervasive effects of an antibiotic on the human gut microbiota, as revealed by deep 16S rRNA sequencing.. PLoS Biol.

[38] Riehle KP (2011). The Genboree microbiome toolset for the analysis of 16S rRNA microbial sequences.. BMC Bioinformatics.

[39] Charlson ES, Chen J, Custers-Allen R, Bittinger K, Li H (2010). Disordered Microbial Communities in the Upper Respiratory Tract of Cigarette Smokers.. PLoS ONE.

[40] Ravel J, Gajer P, Abdo Z, Schneider GM, Koenig SS (2011). Microbes and health sackler colloquium: vaginal microbiome of reproductive age women.. Proc Natl Acad Sci.

[41] Dominguez-Bello MG, Blaser MJ, Ley RE, Knight R (2011). Development of the human gastrointestinal microbiota and insights from high throughput sequencing.. Gastroenterology.

[42] Hamady M, Knight R (2009). Microbial community profiling for human microbiome projects: Tools, techniques, and challenges.. Genome Res.

[43] Wang Q, Garrity GM, Tiedje JM, Cole JR (2007). Naive Bayesian classifier for rapid assignment of rRNA sequences into the new bacterial taxonomy.. Appl Environ Microbio.

[44] Cole JR, Wang Q, Cardenas E, Fish J, Chai B (2009). The Ribosomal Database Project: improved alignments and new tools for rRNA analysis.. Nucleic Acids Res.

[45] Whittaker RH (1972). Evolution and measurement of species diversity.. Taxon.

[46] R Development Core Team (2008). R: A language and environment for statistical computing.. Vienna Austria R Foundation for Statistical Computing.

[47] Pridmore RD, Berger B, Desiere F, Vilanova D, Barretto C (2004). The genome sequence of the probiotic intestinal bacterium Lactobacillus johnsonii NCC 533.. Proc Natl Acad Sci U S A.

[48] Kindt R, Coe R (2005). Tree diversity analysis. A manual and software for common statistical methods for ecological and biodiversity studies.. Nairobi: World Agroforestry Centre (ICRAF) ISBN 92-9059-179-X.

[49] Oksanen J (2007). Vegan: community ecology package.. R package version 1.

[50] Tóthmérész B (1995). Comparison of Different Methods for Diversity Ordering.. J Veg Sci.

[51] Ciccarelli FD, Doerks T, von Mering C, Creevey CJ, Snel B (2006). Toward automatic reconstruction of a highly resolved tree of life.. Science.

[52] Gavin DG, Oswald WW, Wahl ER, Williams JW (2003). A statistical approach to evaluating distance metrics and analog assignments for pollen records.. Quatern Res.

[53] Oswald WW, Brubaker LB, Hu FS, Gavin DG (2003). Pollen-Vegetation Calibration for Tundra Communities in the Arctic Foothills, Northern Alaska.. J Ecol.

[54] Lozupone C, Knight R (2005). UniFrac: a new phylogenetic method for comparing microbial communities.. Appl Environ Microbio.

[55] Lozupone CA, Hamady M, Kelley ST, Knight R (2007). Quantitative and qualitative beta diversity measures lead to different insights into factors that structure microbial communities.. Appl Environ Microbio.

[56] Fierer N, Lauber CL, Zhou N, McDonald D, Costello EK (2010). Forensic identification using skin bacterial communities.. Proc Natl Acad Sci USA.

[57] Letunic I, Bork P (2007). Interactive Tree Of Life (iTOL): an online tool for phylogenetic tree display and annotation.. Bioinformatics.

[58] Knights D, Kuczynski J, Koren O, Ley RE, Field D (2010). Supervised classification of microbiota mitigates mislabeling errors.. ISME J.

[59] Manly B (1986). Multivariate statistical method: A primer.. Chapman & Hall Ltd, London UK, UK.

[60] Kursa MB, Rudnicki WR (2010). Feature selection with the Boruta package.. J Stat Softw.

[61] Segata N, Izard J, Waldron L, Gevers D, Miropolsky L (2011). Metagenomic biomarker discovery and explanation.. Genome Biol.

[62] Li M, Wang B, Zhang M, Rantalainen M, Wang S (2008). Symbiotic gut microbes modulate human metabolic phenotypes.. Proc Natl Acad Sci USA.

[63] Price MN, Dehal PS, Arkin AP (2009). FastTree: computing large minimum evolution trees with profiles instead of a distance matrix.. Mol Biol Evol.

