# Supplementary figures and images for: A Metagenomic Approach to Characterization of the Vaginal Microbiome Signature in Pregnancy
Source: PLoS One. 2012 Jun 13;7(6):e36466. doi: 10.1371/journal.pone.0036466 (PMC3374618; doi:10.1371/journal.pone.0036466)

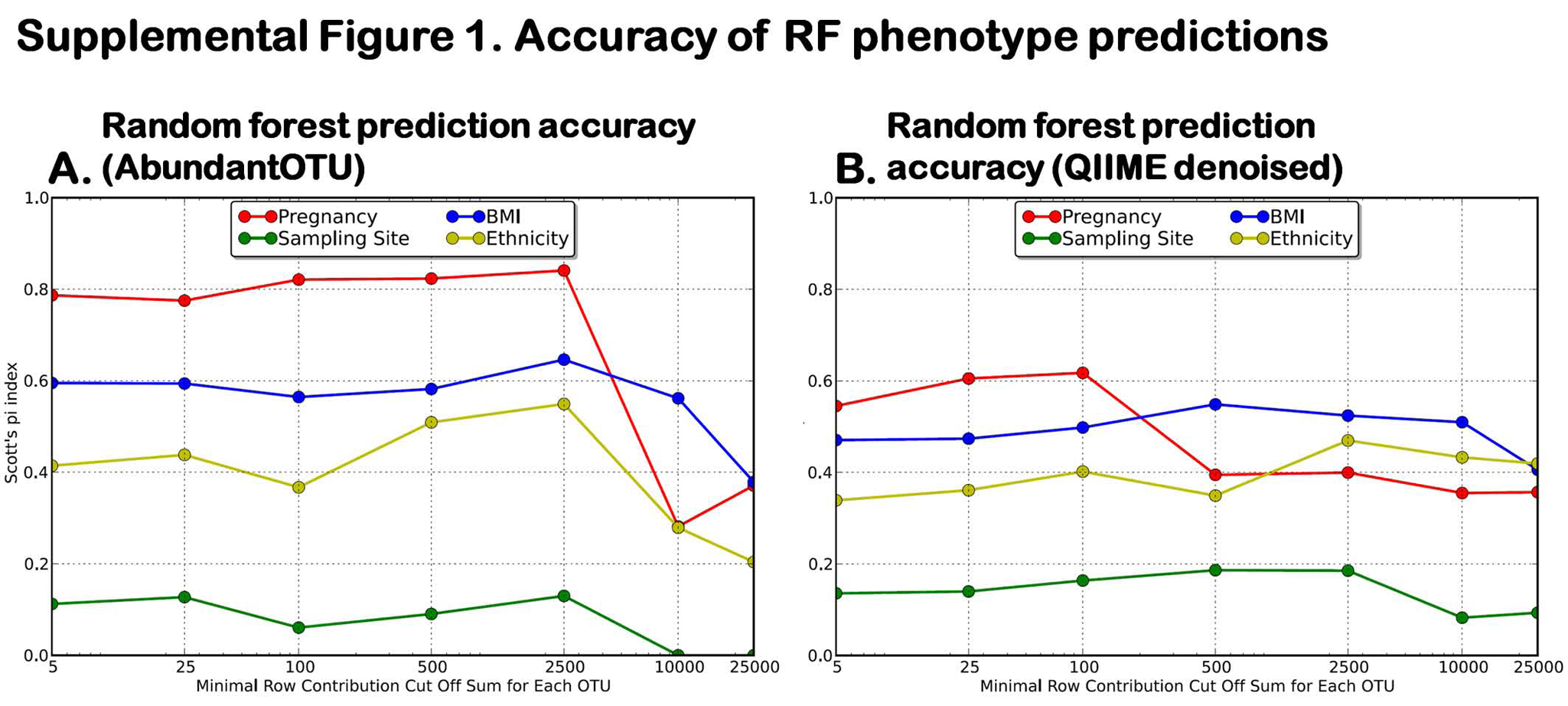

Supplement: Figure S1 — Accuracy of phenotypic predictions from microbiome composition by randomForest. OTU abundances using increasingly many minimal row contributions were used by RF machine learning to predict clinical metadata including pregnancy status, vaginal sampling subsite, subject body mass index, and race/ethnicity. Pregnancy was extremely well predicted by OTU features (Scott’s pi >0.8, as compared to a random baseline of 0), exceeding the (still high) accuracy of other metadata predictions. (TIF) [file pone.0036466.s001.tif]

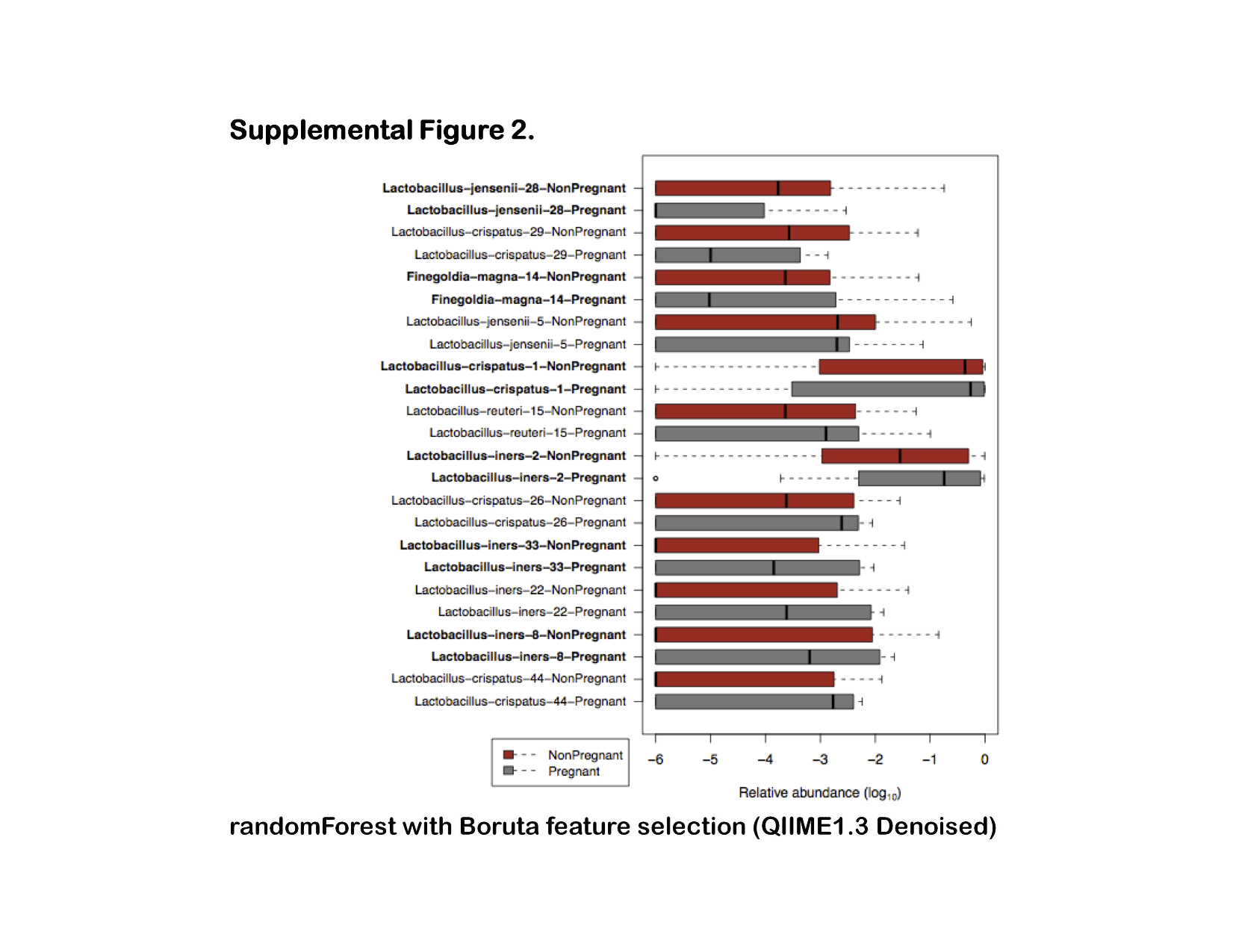

Supplement: Figure S2 — Supervised (machine) learning with definition by randomForest and confirmation by Boruta feature selection enables visualization of bacterial taxa contributing to clustering of vaginal communities in pregnancy. Bacterial taxa (leftmost column) were defined by randomForest (Table S1) and confirmed by Boruta feature selection. Taxa are sorted first by Mann-Whitney U score, followed by the largest disparity in medians for each group. Taxa represent the lowest taxonomic depth (Genus) that are labeled by RDP Classifier (at ≥80% bootstrap cut off). Boxes represent the first quartile, median, and third quartile of the distribution of OTUs for each sample group. Empty circles represent outliers that are 1.5-fold greater than the respective interquartile ranges. (TIF) [file pone.0036466.s002.tif]

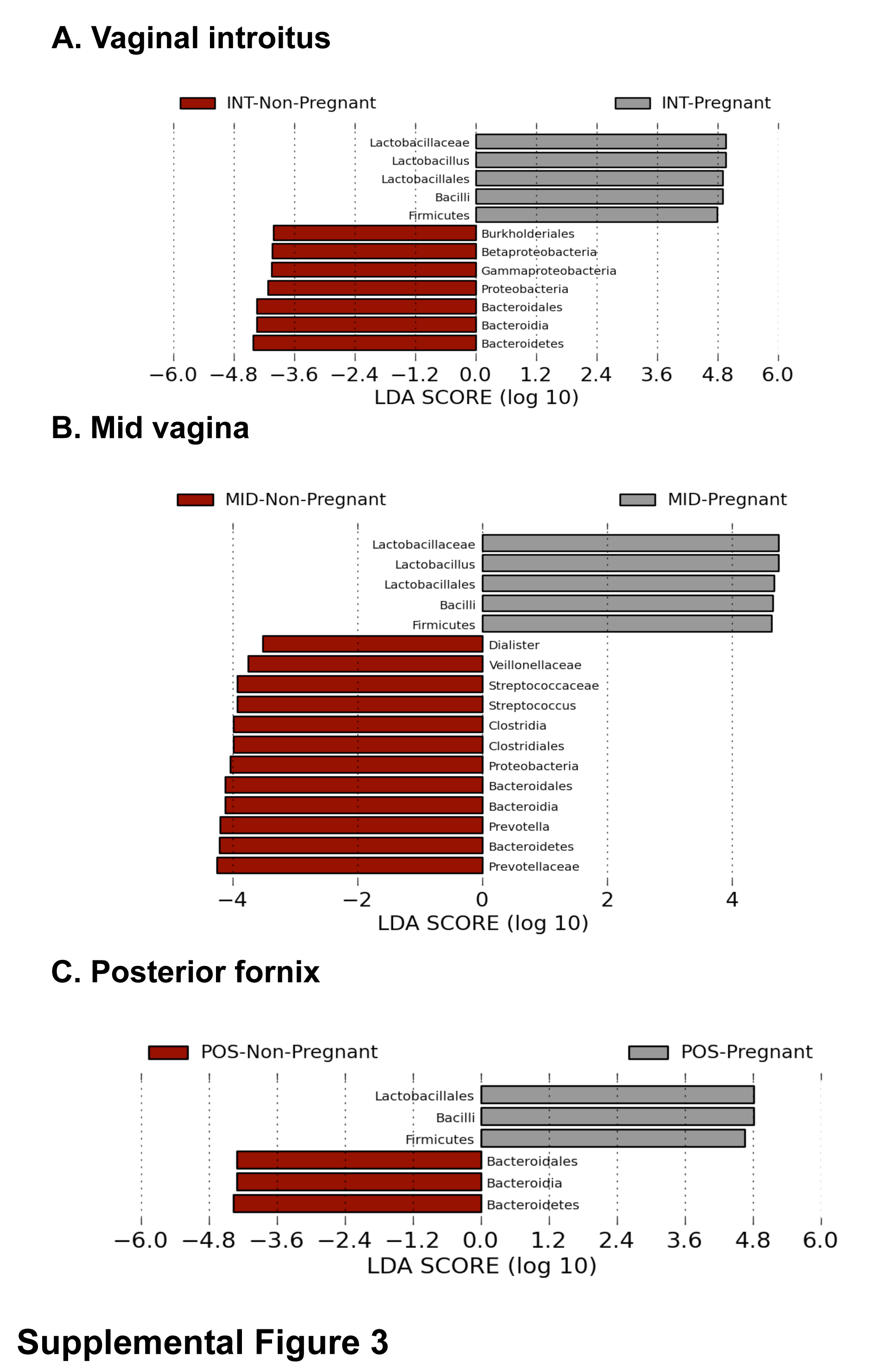

Supplement: Figure S3 — LEfSe analysis of binned taxonomy at discrete vaginal subsites. Bacterial taxa were selected as significantly differentially abundant between pregnant and non-pregnant communities by virtue of discrete sampling site and displayed by LDA Effect Size (LEfSe) algorithm. Taxa level projections are defined by pregnancy at each subsite, with specific Lactobacillus species detected consistently among pregnant individuals. (TIF) [file pone.0036466.s003.tif]
