# Supplementary material for: A Metagenomic Approach to Characterization of the Vaginal Microbiome Signature in Pregnancy
Source: PLoS One. 2012 Jun 13;7(6):e36466. doi: 10.1371/journal.pone.0036466 (PMC3374618; doi:10.1371/journal.pone.0036466)
Supplement: Table S1 — Estimated error rate of the randomForest simulation by virtue of potentially contributable clinical metadata (pregnancy, BMI, vaginal sampling site, and ethnicity) following abundant OTU pipeline for denoising of dataset. Top row header: Minimal row contribution cut off sum for each OTU to determine the best performing data set (i.e., contains the most discriminative features with least amount of noise). When describing estimated error rate per minimal row contribution, pregnancy was retained as the only significant clinical metadata category in the model simulation that had an acceptable level of estimated error (<10%, in bold face type). (DOC) [file pone.0036466.s004.doc]

| **Supplemental Table 1. Supervised (machine) Learning Estimated Error Rates (randomForest simulation) for abundantOTU denoised pipelines.** | | | | | | | |
| --- | --- | --- | --- | --- | --- | --- | --- |
| **Clinical characteristic** | **Minimal Row Contribution Cut Off Sum for Each OTU** | | | | | | |
|  | **5** | **25** | **100** | **500** | **2,500** | **10,000** | **25,000** |
|  | **Estimated Error Rate (percent, %)** | | | | | | |
| **Pregnancy** | **5.69** | **5.96** | **4.88** | **4.88** | **4.34** | 16.26 | 14.36 |
| **BMI** | 21.41 | 21.41 | 23.04 | 21.95 | 18.97 | 23.58 | 33.33 |
| **Vaginal sampling site** | 58.81 | 57.72 | 62.33 | 60.43 | 57.72 | 66.94 | 74.25 |
| **Ethnicity** | 14.63 | 14.36 | 15.45 | 13.28 | 12.2 | 18.7 | 21.68 |
