# Supplementary material for: A Metagenomic Approach to Characterization of the Vaginal Microbiome Signature in Pregnancy
Source: PLoS One. 2012 Jun 13;7(6):e36466. doi: 10.1371/journal.pone.0036466 (PMC3374618; doi:10.1371/journal.pone.0036466)
Supplement: Table S3 — The OTUs selected by Boruta feature selection are assigned to the species level by realigned to Greengenes database and BLAST with NCBI microbial database. Top OTUs identified by LEfSe are marked with an asterics. (DOC) [file pone.0036466.s006.doc]

| **Top OTUs Confirmed by Boruta (AbundantOTU)** | **Species** |  |  |
| --- | --- | --- | --- |
| Consensus2 | Lactobacillus iners | | * |
| Consensus102 | Lactobacillus crispatus | | * |
| Consensus84 | Lactobacillus crispatus | | * |
| Consensus70 | Lactobacillus crispatus | | * |
| Consensus98 | Lactobacillus crispatus | | * |
| Consensus67 | Lactobacillus crispatus | | * |
| Consensus44 | Lactobacillus crispatus | | * |
| Consensus33 | Lactobacillus iners | | * |
| Consensus29 | Lactobacillus crispatus | | * |
| Consensus46 | Lactobacillus crispatus | | * |
| Consensus28 | Lactobacillus jensenii | | * |
| Consensus1 | Lactobacillus crispatus | |  |
| Consensus111 | Lactobacillus iners | |  |
| Consensus115 | Lactobacillus crispatus | |  |
| Consensus117 | Anaerococcus lactolyticus | | |
| Consensus118 | Lactobacillus iners | |  |
| Consensus14 | Finegoldia magna | |  |
| Consensus15 | Lactobacillus reuteri | |  |
| Consensus22 | Lactobacillus iners | |  |
| Consensus24 | Prevotella bivia | |  |
| Consensus25 | Lactobacillus jensenii | |  |
| Consensus26 | Lactobacillus crispatus | |  |
| Consensus27 | Fusobacterium nucleatum | | |
| Consensus3 | Lactobacillus johnsonii | |  |
| Consensus30 | Burkholderia cenocepacia | | |
| Consensus32 | Lactobacillus johnsonii | |  |
| Consensus35 | Dialister invisus | |  |
| Consensus37 | Lactobacillus johnsonii | |  |
| Consensus39 | Lactobacillus johnsonii | |  |
| Consensus4 | Gardnerella vaginalis | |  |
| Consensus42 | Ureaplasma parvum | |  |
| Consensus43 | Lactobacillus johnsonii | |  |
| Consensus48 | Lactobacillus crispatus | |  |
| Consensus5 | Lactobacillus jensenii | |  |
| Consensus53 | Streptococcus anginosus | |  |
| Consensus58 | Bacteroides ureolyticus | |  |
| Consensus59 | Lactobacillus crispatus | |  |
| Consensus60 | Stenotrophomonas maltophilia | | |
| Consensus61 | Corynebacterium lipophiloflavum | | |
| Consensus7 | Atopobium vaginae | |  |
| Consensus71 | Catonella morbi | |  |
| Consensus73 | Lactobacillus coleohominis | | |
| Consensus8 | Lactobacillus iners | |  |
| Consensus86 | Atopobium vaginae | |  |
| Consensus90 | Lactobacillus crispatus | |  |
| Consensus92 | Lactobacillus jensenii | |  |
| Consensus94 | Lactobacillus johnsonii | |  |
| Consensus96 | Lactobacillus iners | |  |
| Consensus97 | Lactobacillus johnsonii | |  |
|  |  |  |  |
| **Top OTUs Confirmed by Boruta (QIIME)** | **Species** |  |  |
| 882 | Lactobacillus johnsonii | | * |
| 322 | Lactobacillus crispatus | | * |
| 351 | Bacteroides vulgatus | |  |
| 354 | Gardnerella vaginalis | |  |
| 302 | Burkholderia cenocepacia | | |
| 625 | Anaerococcus lactolyticus | | |
| 125 | Finegoldia magna | |  |
| 301 | Anaerococcus prevotii | |  |
| 714 | Atopobium vaginae | |  |
| 337 | Corynebacterium lipophiloflavum | | |
| 885 | Corynebacteriumamycolatum | | |
| 1084 | Lactobacillus johnsonii | |  |
| 974 | Lactobacillus crispatus | |  |
| 129 | Lactobacillus coleohominis | | |
| 411 | Lactobacillus jensenii | |  |
| 1093 | Lactobacillus iners | |  |
| 57 | Lactobacillus reuteri | |  |
| 39 | Ureaplasma parvum | |  |
| 1054 | Prevotella timonensis | |  |
| 795 | Prevotella amnii | |  |
| 210 | Prevotella bivia | |  |
| 423 | Streptococcus anginosus | |  |
| 603 | Dialister micraerophilus | |  |
| 360 | Veillonella atypica | |  |
| 1028 | Dialister invisus | |  |
| 307 | Stenotrophomonas maltophilia | | |
